# Split-Second Unlearning: Developing a Theory of Psychophysiological Dis-ease

**DOI:** 10.3389/fpsyg.2021.716535

**Published:** 2021-11-29

**Authors:** Matt Hudson, Mark I. Johnson

**Affiliations:** ^1^Mind Help Limited, Durham, United Kingdom; ^2^Centre for Pain Research, Leeds Beckett University, Leeds, United Kingdom

**Keywords:** psychological trauma, physiological stress, psychophysiological dis-ease, emotional memory image (EMI), hypothalamic-pituitary-adrenal (HPA) axis, talking therapies, psychological therapies focused on trauma, split-second unlearning

## Abstract

Psychophysiological “stress” underpins many conditions including anxiety, depression, phobias, chronic fatigue syndrome and non-specific musculoskeletal pain such as fibromyalgia. In this article we develop an understanding of chronic psychophysiological stress from a psychological educational perspective, by drawing on supporting evidence that significant emotional events in early life (traumatic and benign) can influence health and well-being later in life. We suggest that traumatic events instigate psychophysiological “stress” responses and the formation of emotional memory images (EMIs) within very short time frames, i.e., “split-second learning.” Once formed these emotional memories are triggered in daily living “re-playing” psychophysiological stress responses, resulting in chronic psychophysiological “dis-ease.” We describe a novel therapeutic approach to scan clients for mannerisms signifying a subconscious “freeze-like” stress response that involves the client as a curious observer within their own experience, feeding back the non-verbal cues as they arrive in the moment. By breaking down the observable fragments of their split-second Pavlovian response to the trigger, clients can detach their EMI from the psychophysiology stress response, i.e., “split-second unlearning.” Our split-second unlearning model recognizes the EMI as a barrier to moving forward and needs to be unlearned before the client can become naturally adaptive again. We argue that this approach places the client at the center of the work without the need of getting bogged down in a life-long narrative.

## Introduction

Psychophysiological dis-ease is an uneasiness in the mind and deregulation of the hypothalamic-pituitary-adrenal (HPA) axis resulting in psychophysiological “stress.” This underpins many conditions including anxiety, depression, phobias, chronic fatigue syndrome and non-specific musculoskeletal pain such as fibromyalgia. The use of talking-based approaches to address psychophysiological dis-ease has been known about for millennia, particularly among the ancient Greek philosophers (Green et al., 2003). Among these, Hippocrates speculated on the links between physical and psychological health, giving rise to a central wellbeing tenet, “healthy mind, healthy body,” still in use today (Kleisiaris et al., 2014). Post-enlightenment and twentieth century thinkers contributed a great number of perspectives on mental health, bringing these into the medical sphere while also advancing new theories of mind with a view to developing therapeutic frameworks. Freud et al. (1890s –1920s) drew on case studies and classical literature to develop elaborate theories on how the mind is structured, as well as intervention methods that remain significant to this day. The mid-twentieth century saw behaviorism become formalized into a theory to present a more mechanistic explanation of human learning and behavior (see for example work by Watson, 1930; Skinner, 1963, 1980, 1988; Schneider and Morris, 1987). Conversely, humanistic approaches take a more holistic perspective, drawing on human-centered and social theories to develop a more feeling or conscious view of human psychology (see Maslow, 1943; Rogers, 1985). In the later twentieth and early twenty-first centuries, more “cognitive” approaches emerged into the mainstream (see Bandura, 1982; Beck, 1993), challenging the limitations of previous models and enabling a more logic-based study of the psychological phenomena they were supposed to explain. From this emerged Cognitive Behavioral Therapy (CBT), an “evidence-based” approach (albeit within the strictly hierarchical definition of evidence required by the National Institute of Health and Care Excellence (NICE) guidelines) that is comparatively accessible and cheap to implement compared with other psychological therapies. For this reason, CBT has become one of the primary therapeutic approaches used in the UK National Health Service.

Against this backdrop, a large number of more specific theories and models has emerged, many of which have found traction in various therapeutic communities. While the need for evidence-based approaches remains paramount in public mental health policy, those in private practice and some integrative therapists do not have the same types of limitation. They can arguably make use of a wider range of theories, models and approaches. This of course opens the door to pseudoscience but also enables a more fertile ground for new ideas. Such ideas can be esoteric, with varying levels of credibility and passionate detractors and adherents. Broad theories include neuro-linguistic programming (NLP), which borrows from a wide range of disciplines to provide a bespoke “package” of approaches (Bandler and Grinder, 1975); while more specific theories include eye movement desensitization and reprocessing (EMDR), which refines a theoretical framework around observing a specific set of human responses (Feske, 1998; Shapiro and Laliotis, 2015).

Andrade et al hypothesized that the positive effects of EMDR on the symptoms of post-traumatic stress disorder (PTSD) resulted from disruption of the visuospatial sketchpad of working memory. They found that the use of autobiographical stimuli produced a greater reduction in emotional response with EMDR (Andrade et al., 1997). Holmes et al. (2004) have used the “stressful film paradigm,” developed by Lazarus et al. (1965) and Horowitz (1975), to study intrusive mental images in non-clinical participants under controlled settings to provide insights to theoretical and clinical questions. Understanding why certain people create more intrusive images than others is related to how the brain encodes the traumatic experience at the time of the event, with mental images being connected to emotions and anxiety (Holmes et al., 2004; Holmes and Mathews, 2005). For an in-depth review of mental imagery see (Pearson et al., 2015).

These and other esoteric approaches can make the market of psychological theories complicated to navigate. However, we are of the general opinion that any approach which helps a therapist and their client gain a shared insight into the client’s mental processes can be helpful in making therapeutic progress.

It is within this open-minded ethos—and the complication of ideas relating to emotions, mental health, learning, wellbeing and physical health—that we situate the split-second unlearning (SSU) model. Like other theories of mind, the SSU model provides a scaffold to support potential explanations of physical ill health. Drawing on the widely held concept that traumatic (or even apparently benign) events in early life can influence mental health later in life, the SSU model develops that idea to incorporate learned neurological responses, giving rise to physiological symptoms. While it is being explored in several active studies, the core model indicates new therapeutic approaches, including technological solutions, and has important implications for privacy and the necessity for human intervention. This article will describe the SSU model; outline its philosophical foundations; examine its connections with physiological systems; locate it among sibling theories; and speculate on its potential therapeutic uses.

## The Split-Second Unlearning Model

The model combines several psychological and neurological theories to offer a new perspective on the treatment of common mental health issues such as stress and anxiety, as well as more nebulous conditions, such as unexplained pain or fibromyalgia (i.e., chronic primary pain). In brief, SSU proposes that a traumatic past experience is linked with a physiological response. When a person encounters subsequent “reminders” of that experience, consciously or unconsciously, that physiological response is re-triggered. While this might not be as powerful as the first time (more like an “echo” of the original), it is recurrent. Over time, the cumulative effect of this low-level yet persistent physical and psychological stress leads to a wide range of symptoms. If the connection between the trigger memory, or “reminder,” and the response can be severed, then the symptoms of psychophysiological dis-ease may improve. Having the ability to neutralize a traumatic memory and its associated stress response has significant implications for an individual’s allostatic load (Peters et al., 2017) and consequently for a wide range of physical and mental health conditions.

### Underlying Trauma

Traumatic experiences often give rise to a reflexive, unconscious stress response, commonly known as the “fight or flight” response (Cannon, 1939). In the moment, this can be lifesaving; the change in muscle tension, heightened alertness, and redirected blood flow away from non-essential systems enable the subject to rapidly escape from danger. More recent work by Gray (1987) and Bracha et al. (2004) suggests the addition of a “freeze” response, which corresponds with “hypervigilance” and fear (Roelofs, 2017). This is critical in our model, which incorporates ideas around an Emotional Memory Image (EMI), frozen in time and intrinsically linked to a set of physical reactions, including eye movement and/or fixation. In modern life, a traumatic or stress-inducing experience might be connected with more contemporary challenges, such as attending a job interview. These types of scenarios can induce a “fight, flight, freeze” response, in some cases to devastating effect. While it is natural to feel nervous for an interview, there is no survival advantage to be gained from having a dry throat in that situation as the successful interview would lead to a better life. Although, if the interviewee were to have a negative EMI from childhood associated with being in a similar environment i.e., an encounter with a person who has power over the individual, then the autonomic stress response may be appropriate to the context, but not to this specific instance (D’Andrea et al., 2013). Evolutionary psychology might provide a more detailed explanation (Karasewich and Kuhlmeier, 2019) but stress and anxiety, whose distant origins might lie in the realms of natural selection, are more often seen as hindrances in the modern world.

However, a stress response accompanying a specific traumatic past experience at a specific moment in time can be “learned.” Behavioral theory links experiences with either pain or a reward. When a significant event occurs, learning takes place, which eventually crystallizes as a regular, predictable response. It follows therefore that when a person encounters a similar threat or experience, that same response is triggered. The SSU model proposes that repeated triggering of a somatic nervous response, over time, is responsible for a wide range of conditions that diminish everyday wellbeing, including depression and anxiety, stress, and even chronic pain.

Where this model departs from classical psychoanalysis is that the memory need not have been repressed or denied conscious attention. It may be that specific events can be recalled, but the client does not necessarily understand or have conscious awareness of their dramatic physiological response. Our case studies include both fully conscious descriptions of a traumatic moment, accompanied by an unconscious reflexive stress response; and an example of a client who was not conscious of any specific memory but for whom a stress response was clearly being triggered repeatedly by something buried deep in their memory. For Freud, a traumatic event may be repressed or “hidden” from conscious view because it is too difficult to process. However, it remains active in the individual’s unconscious, shaping their character and manifesting as neuroses and consequent unusual behavioral symptoms. Material that is too intense to be consciously processed may become unconscious. For Van der Kolk (1994), this material is inscribed in terms of a physiological response, losing its narrative and instead becoming a reflex bodily expression. The SSU theory subscribes to this position but also allows for clients to be fully aware of the content or narrative of their traumatic memories. The unconscious part of this theory is the learned reflex response that accompanies those memories causing ongoing physical or mental distress.

Following a traumatic event, an “emotional memory image” (EMI) is retained (Bernheim, 2018) and becomes part of a subconscious “danger list” (Lang, 1979; see Ji et al., 2016) for review. External inputs, via any of the senses, that are symbolic of or similar to the original experience, can invoke a specific EMI, triggering the body’s learned stress response. Key to understanding this model is recognizing that the physical response might not be as strong as it was the first time round and might not even be consciously recognized as a response (we learn to ignore involuntary physical reactions, such as ticks, if they happen frequently enough). The cumulative effect of frequently triggered low-key “fight or flight” responses causes a great deal of physical stress, which we propose is enough to underpin a wide range of negative health conditions. These are explored briefly in “Discussion” section.

### Physiological Systems: HPA Axis and Emotional Memory

While some evolutionary-type explanations have been criticized for endorsing a deterministic model of human behavior (Plotkin, 2008), the classic “fight, flight, freeze” theory offers a more complex set of mechanisms. This draws on multiple interactions between physiological, emotional and cognitive systems. The stress response, which encompasses the amygdala and HPA axis simultaneously, plays a key role in a broad range of mental and physical health conditions including psychopathology associated with early adverse experiences (Felitti et al., 1998b; Juruena et al., 2020; El Mlili et al., 2021). This interdependent relationship between the psychological trauma and physiological stress was formally outlined by Selye (1946), referred to as “general adaptation syndrome,” and has since been increasingly refined.

Improvements in knowledge about mechanisms and organization of learning and memory have direct implications for psychopathology (for reviews see Nadel and Hardt, 2011; Pile et al., 2021). The hippocampus has a key role in the processing of working and long-term memory. Learning creates a memory trace, which is activated by a modulation phase essential for memory consolidation and reconsolidation associated with memory stabilization (Alberini, 2005; Alberini and Ledoux, 2013). Stress may enhance or impair memory according to the magnitude of arousal and emotionality associated with the intensity, duration, and context of a traumatic event. During the adaptive stress response consolidation of potentially threatening and dangerous events is given priority and memory retrieval diminished. Stress hormones may facilitate strong and persistent maladaptive or traumatic memories (Drexler and Wolf, 2017; Wolf, 2017).

Retrieval of emotional autobiographical memories involves a network of neural structures in the right-hemisphere that includes connections between the amygdala, hippocampus, and pre-frontal cortex. The amygdala orchestrates activities of structures involved in mediating emotion and memory retrieval but does not necessarily store the unpleasant memories *per se*. Retrieval of an emotional event, cued by a variety of direct or indirect stimuli, involves activation of the amygdala and medial pre-frontal cortex, in a similar manner to the original emotional experience. This results in an emotional state and associated autonomic and somatic responses mediated via neural centers in the hypothalamus and brainstem. Neuropsychology and neuroimaging studies support the amygdala having a key role in the re-experience of an emotional memory. People with damage to the medial temporal lobe affecting both the amygdala and the hippocampus recollect fewer unpleasant autobiographical memories, with the right amygdala playing a role in the retrieval of unpleasant, intense autobiographical experiences irrespective of the integrity of the left amygdala. However, people with damage limited to the hippocampus are able to recall autobiographical memories of emotional events suggesting that emotional memories are not fully dependent on the hippocampus (for review see Buchanan, 2007).

Behavior modulation is paramount to survival and inappropriate communication could lead to detrimental responses. The amygdala has a role in motivational drive or lack of Cunningham et al. (2010) and our SSU model pays particular attention to the role of the amygdala in the onset of psychophysiological dis-ease. We hypothesize that a client may be held within an “amygdala trap” in the form of a freeze-like survival response because they are unable to fight or flee from their original adverse experience, manifesting over time as poor mental and/or physical wellbeing (Roelofs, 2017). Whilst the negative EMI appears within the mind’s eye, the client will respond automatically, like the lemming not knowing why the cliff is so compelling. The specific neurophysiological mechanisms within the amygdala associated with such fear-learning are unknown, although theta oscillations appear to have a role (Chen et al., 2021).

We propose that when incoming information matches a stored EMI in any way, then the learned response will trigger the amygdala and HPA axis—even at a low level—to re-enact the original fight, flight, freeze reflex. Although the HPA response is near instantaneous, its physical effects, such as a changed hormonal balance, increased blood pressure, muscle tension etc. take some time to subside. Regular HPA stimulation therefore puts the body through a great deal of stress and has been implicated in a range of psychophysical problems, including systemic inflammation (Powers et al., 2019), adverse immune responses (Antoni and Dhabhar, 2019), sleep disorders (Buckley and Schatzberg, 2005; El Mlili et al., 2021), memory problems (Labad et al., 2020), and major depression (Pariante and Lightman, 2008; Menke et al., 2018). We also speculate that persistent repeated stimulation of this stress response might have a similar effect to “learned helplessness,” which also leads to clinical depression (Miller and Seligman, 1975; Danese and McEwen, 2012).

Moreover, when an EMI triggers the “fight, flight, freeze” response, engaging the amygdala and shutting down the prefrontal lobe, the individual effectively becomes “trapped” in this primitive response state, unable to reason their way out of it. The question then arises: How can the subject free themselves from their problem when the response is involuntary and perhaps even undetectable? Crucially, the HPA response is also associated with parts of the brain that are involved with visual processing. This is shown in several ways, most notably through rapid eye movement (REM) within sleep research (GarcIìa-Borreguero et al., 2000; Buckley and Schatzberg, 2005; Liyanarachchi et al., 2017); ophthalmic studies (Agarwal and Gupta, 2018); and psychosocial research, which directly links HPA activation to eye movement (Herten et al., 2017).

Getting the client to realize that the EMI is a mental representation and not “real” is an important dissociative step (Holmes et al., 2007). In cases that involve processing fearful memories, Tao et al. (2021) found that “explicit fear processing elicited activations at the pulvinar and para hippocampal gyrus, suggesting visual attention/orientation and contextual association play important roles” (p.1). Recalling fearful memories can therefore result in a corresponding eye movement, giving credibility to the theoretical EMI entity and enabling an intervention to focus on visual elements—involuntary eye movement—as a “way in.” Intense emotions can block the hippocampus response (Van der Kolk, 1994), which can impair recollection of an explicit trauma memory. The resulting “implicit” fear processing “elicited more activations at the cerebellum-amygdala-cortical pathway, indicating an “alarm” system…” (Tao et al., 2021). Such an “alarm” response would produce signals, including eye movements linked to unconscious processing of a visual memory or EMI, which a therapist could also analyze.

Interacting with visuospatial working memory can disrupt traumatic memories. James et al. (2015) found that engaging a subject in visuospatial video game play during the reactivation of a trauma memory had a successful impact in destabilizing the episodic memory. Kessler et al. (2020) found that visuospatial computer game play reduced intrusive memories following experimentally induced trauma. Declarative memories are formed by rapid synaptic plasticity in the hippocampus initially encoding transient representations, which are then transferred and consolidated in the neocortex. Gamma (40–100 Hz) and theta (4–8 Hz) oscillations have been noted when an organism is exploring (Bragin et al., 1995; Buzsaki, 2002), yet memory consolidation has been shown to involve the hippocampal neuronal bursts of sharp waves giving high frequency ripple oscillations. These “ripples” correlate to a behavioral measure of memory consolidation (Axmacher et al., 2008, 2009), thus, learning creates a memory trace. The modulation phase is essential for memory consolidation, which is activated by first time learning and memory reactivation (Alberini, 2005; Alberini and Ledoux, 2013). If the modulation process is interrupted after reactivation, then the memory can be impaired. This affords a window of opportunity, during which, the memory becomes labile and can be updated or modified.

## What Is the Process?

**FIGURE 1:**
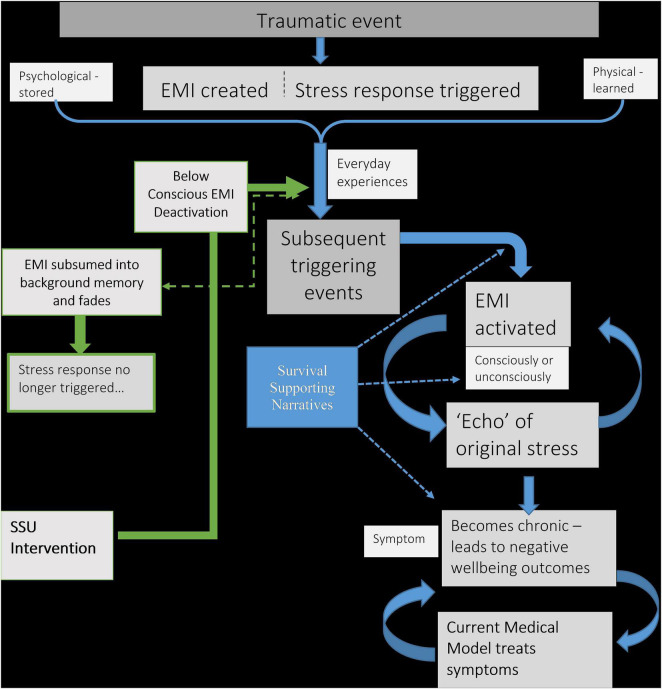
Schematic diagram explaining the connection between traumatic events and chronic negative wellbeing. Also includes proposed intervention. EMI, Emotional Memory Image; SSU, split-second unlearning.

Having described the proposed connection between traumatic events and poor wellbeing outcomes, the following schematic diagram (Figure 1) outlines this mechanism. It also includes the intervention approach, discussed in the next section.

### Intervention

Treatment is based on using open questions in an attempt to prompt the client’s physical HPA axis response and bring this to their conscious attention. The therapist may ask “What would you like to work with today?” or “What is troubling you at the moment?” These questions are designed to provoke the client into scanning their cache of memories in relation to the problem they are seeking to solve. This scan is carried out unconsciously, prior to formulating a conscious verbal reply. When they encounter the troubling EMI, this triggers the negative somatic response, generating the non-verbal signals the therapist is looking for. While the conscious verbal answer may set the scene for psychotherapeutic exploration, their unconscious physical response indicates a distinct connection between a thought and a reflex—between mind and body. This is the connection the SSU practitioner seeks to break.

Within a split-second, the disturbing EMI is re-triggered. Observable effects are subtle but include: a sharp intake of breath, head tilt, muscle tension, pupil dilation and eyes fixating on a specific point in space—all symptoms that are associated with the “fight, flight, freeze” response. Disturbances within the non-verbal channels and non-verbal indicators of emotional distress, such as recalling a traumatic memory, are linked with a wide range of psychological and physical conditions (Plusquellec and Denault, 2018). We propose that a range of symptoms and conditions might be addressed by using non-verbal indicators to identify the source of distress.

Among these non-verbal indicators, eye fixation is critical. In the SSU model, staring at a specific point in space indicates the subject is recalling a specific memory and a specific point in time. This may be related to the content of the EMI—for example, a subject who has a fear of heights may look down; with agoraphobia, they might search the periphery of their visual field; or if a specific event is being recalled, the client may look in the direction in which they initially encountered the threat. More complex traumatic issues might yield no obvious interpretive correlation between the focal point and the memory, other than the same point is fixated upon every time that EMI is triggered. There is some evidence to back this up. The client is clearly looking at something; Shapiro (1989) was the first to realize that eye movement is connected with recalling a traumatic event. Subsequent research in Eye Movement Desensitization and Reprocessing (EMDR) has shown that that the scan path of the client’s eyes is revivifying an episode that was set during the original coding of the event (Brandt and Stark, 1997). Johansson et al. (2019) build on this by suggesting that the memory is held in a position and the eyes show “*an order, direction, shape, length and duration.*” In this split second, the subject appears to be observing and re-experiencing their past.

Our intervention technique owes a great deal to EMDR. By examining the client’s eye movements, making eye movement suggestions based on prior EMDR theory, and through careful probing, the EMDR practitioner explores with the client their emotional issues, including triggers and potential sources, working to “clear” or disrupt a distressing emotional state. Through multi-stage exploration, the overall experience is one of learning; the client’s psyche is said to “metabolize” the source of the emotional trauma (Shapiro, 2017). The techniques involved in EMDR include both skilled psychotherapeutic work with the subject, to uncover the underpinning traumatic event, and deep knowledge of specific EMDR theory and practice, updated most recently in Shapiro and Forrest (2001). Similarly, SSU is based on examining a client’s eye movements during a physical stress response, triggered by an EMI (which may represent a “real” object or be linked to a concept). In contrast to EMDR, the SSU practitioner identifies the exact point in space and time at which the traumatic image is evoked then aims to “interrupt” this recollection. The effect is immediate.

### Interruption

The “split second” refers to the almost instantaneous amount of time it takes for the body to “learn” the initial stress response and “replay” it in future situations. Research by Haesen et al. (2017) provides evidence of fast specific fear learning as part of adaptive defensive actions in humans. The “split second” also refers to the brief window the practitioner has in which to observe the activation of the EMI and to deploy a well-timed interruption that will disrupt the association between the memory and the neurological response. The challenge to conventional approaches to trauma and mental/physical health conditions is the time and finance allotted to treatment and recovery. The SSU model is predicated around a single brief physical event, which becomes the focus of the intervention and can be solved in a far shorter amount of time. Encouraging clients to share and explore their experience to gain more insight makes little sense during this type of automatic response, in which a more primitive “fight, flight, freeze” reflex becomes dominant over higher-order functions such as careful reflective reasoning. The subject is now a victim of their EMI.

#### Stage One. The Client Is Made Aware of Their Involuntary Physical Response

As described, the therapist asks a prompt, or set of prompt questions and observes the client’s physical responses prior to them answering verbally. This might be repeated to ensure that the same reflex is being triggered and any specific contextual cues that might be involved at this stage. The therapist then points out to the client the process that they have just gone through before they replied to the question. Often, the client does not notice or is unaware of their process, so the therapist can ask again “what would you like to work with today?” pointing out to the client their automated response to the question.

#### Stage Two. The Therapist Asks the Client to Direct Their Gaze to a Different Position While Still Trying to Think About Their Problem

Focusing on a different point in space while attempting to recollect the subject matter disrupts the visual element of the previously automated stress response produced by the “learned” interaction of the EMI, visual cortex and HPA axis. The method draws on an applied phenomenological communication approach (Arnett, 1981), described within the Case Studies in section “Case Studies.”

The physical response is usually subconscious, and the client is often unaware of what exactly has changed, or why they do not feel the same way. Their memory remains—either consciously or unconsciously but its connection with a physical response is broken. The effect can be felt immediately and often manifests as confusion. However, the long-term effect, resulting from the HPA axis no longer being re-triggered, is more profound. The body has a chance to “heal”; the stress response returns to normal frequency levels, and wellbeing is increased.

Timing is crucial. Interrupting the process during that split-second and making the subject more aware of their non-verbal responses to the EMI enables new learning to take place. When the would-be victim of their EMI is moved toward the position of curious observer, outside of the original event, the “fight, flight, freeze” response does not “kick in” and instead, higher-order cognitive processes take priority. In this moment, it is possible to learn a new response, in the present, with the guidance of a therapist. Gruber et al. (2014) presents compelling experimental evidence that “states of curiosity” enhance our capacity for learning new information, including learned responses. During an SSU therapy session, a new learned response, one of dispassionate acceptance, breaks the previous association between the EMI and the stress response—which no longer serves a useful purpose—replacing it with a more objective appraisal of the overall situation. The EMI may now be infused with a clarity of hindsight, or it might simply be deemed unimportant.

### Presence of a Therapist

Where SSU differs from EMDR is that it focuses on linked physiological responses such as “fight-flight-freeze,” or more chronic stress responses such as heart rate variability. This removes the need for a practitioner to gain deep understanding of the content of the image or to pry into a client’s psyche, which can be perceived as intrusive. Indeed, this type of probing exploration is one the reasons some people are reluctant to undergo psychodynamic psychotherapy. Instead, a client’s physical metrics can be monitored using commercially available devices (e.g., Fitbit, Garmin or Apple Watch). Eye movement can be observed by a therapist but can also be tracked to a high degree of accuracy using mobile phone-based software. One of the criticisms of EMDR has been the increasing complexity and cost of its training programs. Over-commercialization of EMDR training and registration has led to some skepticism and, while such criticism is common around many new approaches to wellbeing personal development, the SSU model offers a fast, simple solution with only limited scope for licensing and commercialization.

We are exploring a version of this approach that does not require the presence of a trained therapist. The Mind Reset app uses software developed by UMoove, an Israeli start-up that specializes in a range of eye-tracking applications, from gaming to health. The app aims to detect regular eye movement patterns while asking the user open-ended questions. Once a pattern has been detected, the app deploys different questions, or distraction techniques, whenever the user’s eyes fall into that pattern. The possibility of “removing the therapist from the room” would be a significant advantage of SSU over other therapeutic approaches. The potential to bypass the need for intrusive psychological probing and expensive training has major implications for the application of this theory in a more refined form.

As a general framework, SSU enables several advantages over current therapeutic approaches, mainly: (a) the intervention is instantaneous; it does not require repeated sessions or long courses of therapy; (b) there is scope, by incorporating eye-scanning technology, to remove the therapist from the picture altogether.

## Discussion

The SSU model draws on a range of ideas from different disciplines, configured into a cohesive framework that connects trauma, stress and intervention. This article describes our first iteration of the model and describes its application, although the range of examples and perspectives is limited. Further theoretical and empirical work would enable theory refinement, evidence generation, and more perspectives for discussion. In this section, we explore specific elements that we find interesting and that reveal more of the ethos from which SSU was developed.

### Ontological Status of the Emotional Memory Image

Twentieth century debates around realist and anti-realist/idealist positions have had a significant influence on philosophy and the social sciences. “Scientific realism” (see Putnam, 1982; Boyd, 1983) is the idea that scientific progress can be made through discussion of unobservable (i.e., theoretical) entities and that the best theories generated from these are the closest we can get to a true approximation of reality. “Entity realism” (Cartwright, 1983; Hacking and Hacking, 1983) emerges from this school, proposing that theoretical entities, subatomic particles for example, definitely exist if they can be manipulated to produce an observable effect. While empirical evidence of some particles remains scant, experiments can be designed to produce observable effects that rely on the existence of those theoretical particles, meaning that they can essentially be considered real.

The experiences of the author and other practitioners indicate that clients locate their EMI in a spatial location outside of the body that therapists can interact with. Through careful manipulation, a practitioner can remove, disrupt or otherwise render harmless a client’s EMI. For this reason, we consider the EMI to be a real entity—a position also endorsed by Nanay (2019). The images we hold about an event or memory, including EMIs, are often deemed constructs—representations of an idea, shaped by our general perceptions of the world. While EMIs might be formed in a specifically filtered way (especially during childhood, when the world is a very different place), the SSU model nevertheless treats these as real, fixed entities that can be manipulated. For example, in case study one (see “Case Study” section), the EMI of the father “turning white” may not be an exact facsimile of what the father looked like—but the EMI itself is nevertheless real and became associated with a real physiological effect.

### Prior Trauma Inscribed Into Somatic Responses

Psychodynamic theory famously promotes the idea that early experiences contribute to the formation of our adult characters and to consequent psychological wellbeing. The SSU model works on the same general premise but incorporates a critical physical component (HPA axis and the stress response). In this respect, the model bridges between its psychotherapeutic forbears and more recent theories, most notably Van der Kolk’s (1994) idea that victims of psychological trauma may exhibit bodily expressions of that traumatic memory. Van der Kolk goes further, explaining that trauma victims might be unaware of a specific memory and/or that a traumatic event has even taken place. Instead, they keep a record through physiological changes in the body and brain, which can predispose a diverse range of physical disorders. It is further claimed that traumatic memories can be “entirely organized on an implicit or perceptual level, without an accompanying narrative about what happened” (Van der Kolk and Fisler, 1995). Rothschild (2000) sums it up succinctly: “The body remembers even if the mind cannot.”

The general idea is seductive, in that it allows for new therapeutic approaches that interpret physical gestures to reveal implicit memories of dissociated trauma. However, McNally (2005) argues that a lack of evidence means that this line of reasoning and the “recovered memory therapy” it inspired is “arguably the most serious catastrophe to strike the mental health field since the lobotomy era.” Nevertheless, while the causal mechanisms remain debatable, there is a growing body of research into the relationships between past psychological trauma and physical symptoms. Research on Adverse Childhood Experience (ACE) also supports the claim that early traumatic events can generate recurrent negative symptoms later in life. Such experiences, including physical and sexual abuse, or neglect, are cited as a predictor of a wide range of physical health conditions (Felitti et al., 1998a). In particular, these include generalized stress dysregulation (Evans and Kim, 2007), which can manifest in a wide range of negative wellbeing outcomes. While childhood experiences can be especially profound for various reasons, we propose that traumatic events experienced at any age can lead to a persistent somatic response. Research into the effects of ACEs is ongoing, and the catalog of physical symptoms these cause undergoes regular revision (Finkelhor et al., 2015).

### Removing the Therapist

EMDR (Shapiro, 1989; Shapiro and Forrest, 2001; Shapiro and Laliotis, 2015) has drawn on empirical evidence to develop a specific therapeutic approach, with its own significant following (notably endorsed by Van der Kolk). While SSU shares some of the diagnostic and intervention elements of EMDR, it rests on a different set of philosophical and psychological underpinnings. One of the criticisms of EMDR is the amount of time and money it takes to qualify as a practitioner (Rosen et al., 1999). Instead of relying on significant interpretation and psychotherapeutic guidance from a trained practitioner, SSU takes a more mechanistic approach, focusing on the connection between a memory (as an entity) and a physical response, rather than the symbolic content of that memory. SSU removes the need for extensive training or psychotherapeutic experience; the approach is altogether more accessible for practitioners, for whom the skill lies in identifying and recognizing a response, rather than in interpretation of content or meaning. While the SSU model shares some common ground with EMDR, its ultimate aim is to gradually “remove the therapist from the room.” This approach moves away from the traditional paternalistic model of western medicine. This returns power to the patient by helping them identify their own subconscious issues and nudging them toward solving these.

Our SSU model presupposes that the client has been exposed to an adverse childhood experience. A negative EMI stored inside the client’s mind is influencing their decisions and impacting their psychophysiological wellbeing. The SSU model does not rely on interpretation of any memory content—merely on close observation and deploying an appropriate intervention with precision timing. These are tasks that can theoretically be undertaken by a machine (such as a mobile phone), giving SSU a significant advantage over other forms of therapy. Notably, removing the need for the “content” of a memory to be analyzed by another person might make this approach far more attractive and accessible for some clients. We are conducting ongoing research using a mobile phone app, which combines eye-tracking scan path data with other biofeedback data to detect eye movement patterns and a stress response. The app then deploys screen-based prompts at specific times to attempt to disrupt the learned response. The challenge of using an app is achieving user engagement when there is an absence of face-to-face therapist-client interaction. At present, the app is introduced within a therapy session, so that the therapist can explain the SSU theory and the app to the client who then uses the app between clinical visits. Studies are underway to see the effect of “removing the therapist from the room” over time.

### Memory, Learning, and Ethics

Research into memory reconsolidation interference has explored the effects of encouraging a subject to re-access their traumatic memory, recreating the same neurobiological processes that were present within the original consolidation (Merlo et al., 2015). This reactivated state increases neuroplasticity, creating the opportunity for an intervention to take place. It has been shown to be possible to create a beneficial impact using memory reconsolidation interference regardless of how many years the subject has suffered with the memory (Beckers and Kindt, 2017).

Given the potential significance of this research, it is vital to consider ethical implications. Is “nudging” or prompting someone in a certain direction just another way of exerting power over them? Arguably yes. Memories are the foundation upon which a person builds their identity of self and their meaning of life. This might be underpinning psychophysiological stress and dis-ease. Therefore, there are ethical implications to be considered when interacting with a person’s self-narrative (Elsey and Kindt, 2016). Removing the therapist from the relationship and using machine-generated prompts to dissociate the EMI does not resolve this quandary. There is much evidence that people feel better precisely because a trained human expert has listened to their story and helped guide them. Removing human interaction from a therapeutic relationship may raise concern and something we are mindful of in our ongoing research.

## Conclusion

In this article we propose a new theory that outlines a mechanism connecting prior traumatic experiences with chronic psychophysiological stress, resulting in dis-ease. This gives rise to an intervention that disrupts or breaks the connection between a memory and a physical symptom in a “split second.” This has immediate effects for the client, bypassing the need to examine the actual content of the traumatic experience. We believe that with some refinement, SSU can be applied to a range of therapeutic scenarios in both physical and mental health domains. Most notably for people suffering with post-traumatic stress disorder and related conditions. While the model is grounded in existing knowledge across disciplines, it requires further testing to generate more robust evidence for its individual components. In future, we hope to investigate patient experiences, long-term outcomes and neurochemical processes (e.g., using functional Magnetic Resonance Imaging techniques).

The need for a scalable, effective, rapid and affordable physical and mental health approach is especially prescient in the wake of the 2020 COVID 19 crisis. We hope that refining the SSU model and therapeutic technique will contribute to alleviating a wide range of trauma-related ailments.

## Case Studies

**FIGURE 2:**
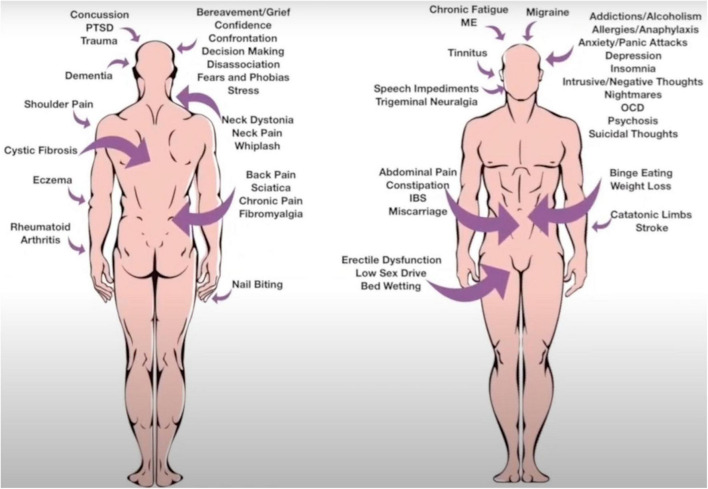
Common conditions managed using the SSU therapeutic approach.

Because SSU does not require extensive or specialist psychotherapeutic training, testing the theory does not require a large investment, so the model has attracted both academic and practitioner interest. One of the authors of this article (MH) is a mental coach and behavioral specialist practitioner. MH was born conductively deaf, which was undiscovered for the first 25 years of his life. This influenced his observation skills, so that he would observe whether the client’s spoken words matched below conscious messages conveyed in behavioral cues expressed during the consultation. This translated into MH taking no notes or case histories, in order that he could be fully present with the client and the client could have full anonymity, knowing that there was no file hidden away with their personal details attached. The SSU model was primarily developed from MH’s extensive experience working one-to-one with clients to resolve a range of psychophysiological conditions including phobias, chronic pain, anxiety, and depression (Figure 2). We describe here two case studies that illustrate the approach in action. The cases are described by MH.

### Case Study One—Male, Aged 51

The client presented with trigeminal neuralgia (chronic pain), which he had been experiencing intermittently for 30 years. Medication made him nauseous. I (MH) asked him to tell me about his pain and the client noted, “it goes back to my bicycle accident,” which he went on to describe in detail. Over the course of about 20 min, I asked things like “what are you noticing, now?” This kept the client aware of the present whilst noticing their experience of the past. Part of the recollection was: “I had a vivid recollection of my father arriving to come and pick me up to take me home. He came into the room, took one look at me, went visibly white and had to leave the room to vomit, then returned.” At this point, the client’s eyes fixated on a particular spot out in front of him, his posture adjusted, he took a sharp intake of breath, the color left his face, and his expression went blank. The total response lasted only a split second. I made a mental note of this action, which quickly faded as the client continued to recount his tale.

#### Intervention

When the client had finished his narrative, I directed him back to the point where he mentioned his father walking into the room. The client re-played all of the aforementioned body cues, suggesting that the experience of his father turning pale and vomiting was traumatic. I asked the client to pretend that he was watching the whole thing on a television over to his right. This would: (a) Have the client fixate his eyes in a different direction to the original EMI; (b) Dissociate the client from the EMI; (c) Allow the client to observe the EMI with me guiding the process; (d) Interrupt the SSU loop, allowing the client to integrate the EMI and move on from it.

The client’s eyes welled up, he breathed deeply, and it was gone. The color returned to his face and he looked a little confused, which I interpreted as the brain reorganizing and updating information—the learned response was being “unlearned.” The stored EMI had left the client repeating the same neurological response over time, preventing any change in experience and perpetuating his pain. Clearing the EMI allowed him to disengage from the survival response, enabling new learning to take place and the split-second to roll forward in time. As the neurological connection with the traumatic memory fades, so do the physical effects of that memory.

The client has not experienced any pain since the intervention in April 2016. During follow-ups, their story has become more general and less detailed, suggesting that the EMI has been subsumed into their everyday background memory, which tends to fade over time, and no longer causes the same physiological problem of chronic pain. In their words: “After the session, I lost the clarity of the pictures of my father coming into the hospital room. I do not seem to be able to retrieve that and much of the vivid detail of some of the experiences of the day of the accident are gone… I had seen my father’s face so clearly for so long but after the session, that memory was different—what remains is a very brief clip of him but no visual detail I can recall of any of the story.”

### Case Study Two—Female, Mid-50s

During a 2-day training course, a lady asked if I would work with her. She shared, to an open audience, that she had been abused as a child by her father. She had completed various forms of therapy and by her own account did not feel traumatized; she had family and a good life. The only problem she had never been able to overcome, was her inability to have regular bowel movements. “Would it be possible for you to help me with this?” she said. As she asked this question, her eyes fixated on a point in her upper-right field of vision. At the same time, she raised her right hand as if to block her gaze.

#### Intervention

I asked her to hold that position then suggested to her that despite all of her years of therapy there appears to be a piece of information that her body had not allowed her to see. I asked her to slowly lower her hand and give herself permission to access the information. At this point, she began to physically tremble, closed her eyes and reported that her brain felt like it was “sizzling with electrical connections.” After a couple of minutes, she opened her eyes, wiped away a tear and said, “thank you.” She could not explain it but knew something had changed. The next morning, in an open round of sharing feedback from the previous day, she raised her hand and exclaimed, “I can poo for Canada!” Having been to the toilet before leaving her house, she was able to have a comfortable natural bowel movement without any effort, which was ground-breaking for her. Her bowel movements remained normal at 6- and 12-month follow-up sessions.

In these interventions, after identifying a set of subtle and fleeting bodily cues, the therapist relied on careful timing to deploy the interruption. Drawing on an applied phenomenological communication approach, commonly used in the caring professions (Bullington et al., 2019; Zahavi and Martiny, 2019), the therapist encouraged the client to focus on the present moment, while simultaneously prompting them to access and reflect upon their traumatic memory. This creates a discrepancy: on one hand enabling the objective witnessing of a memory and its associated reflex response; while on the other, interrupting/preventing the physical cues that cause a client to revisit that memory and subjectively re-experience it. Pragmatically, a well-chosen prompt, directing the subject’s conscious attention to the present, away from their EMI, allows them to become conscious of and objectively experience their stress response. Here, they can engage in actively learning a new (ideally neutral) response, rather than passively experiencing the usual traumatic reflex.

### Experiences of Using the Split-Second Unlearning Model

The SSU model is based on over 20 years of practice and has been used with success to treat phobias, allergies, chronic fatigue syndrome, acute and chronic pain, bedwetting, dyslexia, and addictive behaviors (Figure 2). Often benefit can be achieved within a single session. The underlying premise in the application of the SSU model is to focus on the process of the client communication and not the content. The client is out of rapport with themselves, and the task of the therapist is to remove the negative EMI, so that the client can be in balance again.

The SSU model has a humanistic ethos; the client is not “broken” and therefore cannot be “fixed.” The client’s perception of the world is closed by contextual beliefs and needs to be opened to the infinite possibilities a change in perspective can bring (Cohen, 1986; Firat and Venkatesh, 1993; Schiffrin, 1994). Curiosity, light-heartedness and the ability to constantly reframe the client’s communication are a precursor to the therapeutic approach. Reframing is a widely adopted psychotherapeutic technique taken from NLP that can alter the way in which the client views their map of reality (see Dilts et al., 1980; Bandler and Grinder, 1982; Dilts, 1999; Neudecker et al., 2014).

Clients are pre-screened to check if they have a clinical diagnosis and are willing to be curious about the approach. If a client presents with pain and no EMI is detected, they are referred. From the outset the therapist is constantly scanning the client’s non-verbal communication, are their eyes fixating on a specific spot, at which point does their breathing shift, whilst speaking are both arms moving, one or none? All of this is fed back to the client to bring their awareness to themselves to develop a deep curiosity of their phenomenological experience (Simione et al., 2017). Although, the EMI is based within the field of mental imagery, not all EMI events are visual images; some are auditory. A skilled practitioner recognizes this and may ask “Can you hear a voice?” or “What did you just hear or say to yourself just then?” Often this is sufficient to help the client become aware of the auditory loop that they are trapped inside.

At the end of a session the practitioner can evaluate change by noticing if the client began by referring to their problem in the present “is,” and now uses the past “was,” or no longer avoids the area where the EMI was stored. This “validation point” allows both the client and the therapist to acknowledge that something has changed, even though it may not be possible to articulate exactly what the change is.

SSU is a specific trauma-based intervention that seeks out arousal responses to EMIs that cause psychophysiological dis-ease. The objective for SSU practitioners is to “sort not support,” although practitioners may also be supportive during the session. This attitudinal driven path can often be at odds with the classically paternal therapeutic approaches that we are culturally accustomed to. This may not be effective for client’s who crave emotional support as SSU is a brief therapy. Sometimes the EMI does not “clear away” during a session, and this is when the spoken word needs to be applied. Some clients require additional consultations if their problem returns and sometimes there is a delay in a client realizing that a “shift” has taken place. For example, some clients leave the consultation believing that therapy had not worked, although at 3-month follow-up they reveal, much to their surprise, that they have been “problem-free.”

The impact of trauma is a unique experience for everyone. The SSU model has the potential for adverse events from the dissociation of trauma as the fear of the unknown can be greater than the known (Carleton, 2016). In this way freedom from the trauma has the potential to be as and if not more traumatic than the actual trauma itself. If for example a client’s early life trauma has led them to choose a certain life partner, the clearing of the EMI may free them from their past, only to leave them feeling trapped in a present. This, although not inevitable, must be considered as one of the many alternative futures, that face clients who have been held captive to their own thoughts for many years.

## Ethics Statement

Written informed consent was obtained from the individual(s) for the publication of any potentially identifiable images or data included in this article.

## Author Contributions

MH contributed to conception of the model and wrote the first draft of the manuscript. MJ contributed to the refinement of the model and all sections of the manuscript. Both authors contributed to manuscript revision, read, and approved the submitted version.

## Conflict of Interest

MH was CEO of Mind Help Limited and declares that the research was conducted in the absence of any commercial or financial relationships that could be construed as a potential conflict of interest. In the previous 36 months MJ has received royalties from Oxford University Press for a book unrelated to the topic of this work, and MJ’s employer has received financial income from GlaxoSmithKline plc and TENSCare Ltd. for expert consultancy services and research grant income from GlaxoSmithKline plc. and the Neuromodulation Society of the United Kingdom and Ireland for projects unrelated to the topic of this work.

## Publisher’s Note

All claims expressed in this article are solely those of the authors and do not necessarily represent those of their affiliated organizations, or those of the publisher, the editors and the reviewers. Any product that may be evaluated in this article, or claim that may be made by its manufacturer, is not guaranteed or endorsed by the publisher.
